# Do segmentation metrics reflect clinical reality? A surgeon-centered evaluation in robot-assisted minimally invasive esophagectomy

**DOI:** 10.1007/s00464-025-12266-3

**Published:** 2025-10-10

**Authors:** Ronald de Jong, Yiping Li, Romy van Jaarsveld, Gino Kuiper, Richard van Hillegersberg, Jelle Ruurda, Josien Pluim, Marcel Breeuwer, Yasmina Al Khalil

**Affiliations:** 1 Department of Biomedical Engineering, Eindhoven University of Technology, Groene Loper 3, 5612 AE Eindhoven, The Netherlands; 2 Department of Surgery, University Medical Center Utrecht, Heidelberglaan 100, 3584 CX Utrecht, The Netherlands

**Keywords:** Anatomy recognition, Deep learning, Evaluation metrics, Robot-assisted surgery, Semantic segmentation, Survey

## Abstract

**Background:**

Deep learning-based anatomy segmentation holds promise for improving real-time guidance in complex surgeries such as robot-assisted minimally invasive esophagectomy (RAMIE). However, the clinical relevance of commonly used metrics for evaluating segmentation quality remains unclear, as previous assessments have lacked direct input from surgeons. This study aims to assess how well quantitative segmentation metrics reflect surgeons’ assessments of anatomical overlay accuracy and clinical usefulness during RAMIE.

**Methods:**

We conducted a survey involving 26 upper gastrointestinal surgeons, including both trainee and attending surgeons, who assessed video clips of RAMIE procedures featuring deep learning-generated anatomical overlays. We correlated the surgeons’ qualitative evaluations of annotation accuracy and clinical usefulness with a comprehensive set of quantitative metrics, including overlap, distance, temporal, and error-specific measures. The analysis encompassed over 8000 manually annotated frames from 12 video clips, with overlays generated by two state-of-the-art deep learning models.

**Results:**

Overlap and temporal consistency metrics show the strongest correlation with surgeon assessments. Distance-based and error-specific metrics correlate moderately. Novices show weaker correlations and tend to rate overlays more leniently. Qualitative feedback reveals issues like hallucinations and instability, often missed by current metrics.

**Conclusion:**

Standard quantitative metrics partially reflect surgeon perceptions but should be complemented by surgeon-informed evaluations and task-specific metrics to better capture clinically relevant errors. Aligning metric design with surgical expertise is essential for the safe and effective integration of AI-guided anatomical segmentation in the operating room.

**Graphical abstract:**

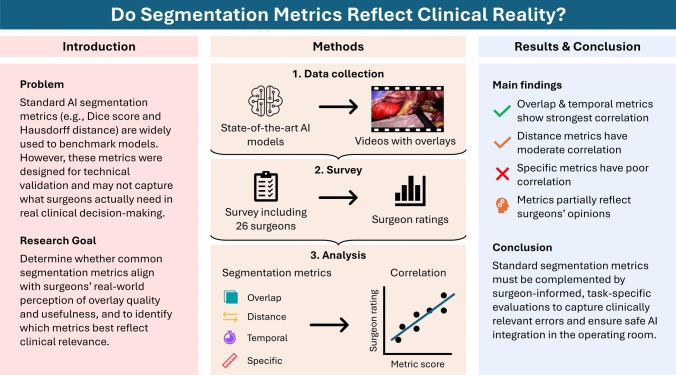

The integration of artificial intelligence into surgical practice has opened new frontiers for enhancing procedural safety, accuracy, and outcomes. Among the most promising applications is deep learning-based anatomy segmentation, which enables real-time identification of critical structures during surgery [1, 2]. Such capabilities are particularly valuable in robot-assisted minimally invasive procedures, where the view is limited and surgical landmarks are often difficult to identify.

Robot-assisted minimally invasive esophagectomy (RAMIE) is a prime example of a complex, high-stakes procedure where anatomical guidance could make a substantial difference. RAMIE involves intricate dissection near critical structures such as the recurrent laryngeal nerves and the thoracic duct, injury to which can lead to complications such as vocal cord paralysis and chylothorax, respectively [3]. These challenges are further compounded by significant anatomical variability between patients. The steep learning curve associated with RAMIE makes it especially difficult for novice surgeons to develop proficiency [4–6]. In this context, real-time anatomical overlays generated by deep learning models have the potential to improve intra-operative orientation, reduce the risk of complications, and accelerate surgical training.

Despite rapid advancements in deep learning models for anatomy segmentation, evaluation has largely relied on standard quantitative metrics such as the Dice score and Hausdorff distance. While these offer objective performance measures, their clinical relevance remains uncertain, particularly regarding how well they align with surgeons’ perceptions of accuracy and practical usefulness during surgery. Most prior studies have emphasized algorithmic performance without integrating direct feedback from surgeons [7–9]. However, surgeon expertise is essential for assessing whether model outputs truly enhance surgical navigation and decision-making [10–12]. Without this input, high metric scores may not translate into meaningful improvements in the operating room.

In this study, we aim to bridge this gap by assessing the clinical relevance of deep learning–based anatomical overlays in RAMIE. Specifically, we correlate commonly used segmentation metrics with qualitative feedback from trainee and attending surgeons to determine how well these metrics reflect practical usefulness and accuracy during surgery. This work provides insights into the strengths and limitations of current evaluation approaches and highlights opportunities for improving both surgical guidance and training.

## Materials and methods

We designed a survey in which surgeons evaluated deep learning–generated overlays in recordings of RAMIE procedures. We combined surgeon feedback with quantitative performance metrics, allowing us to directly compare objective algorithmic measures with subjective clinical assessments. In the sections below, we describe the survey design, participants, deep learning approach, overlay generation, metrics, and evaluation.

### Survey design

**Fig. 1:**
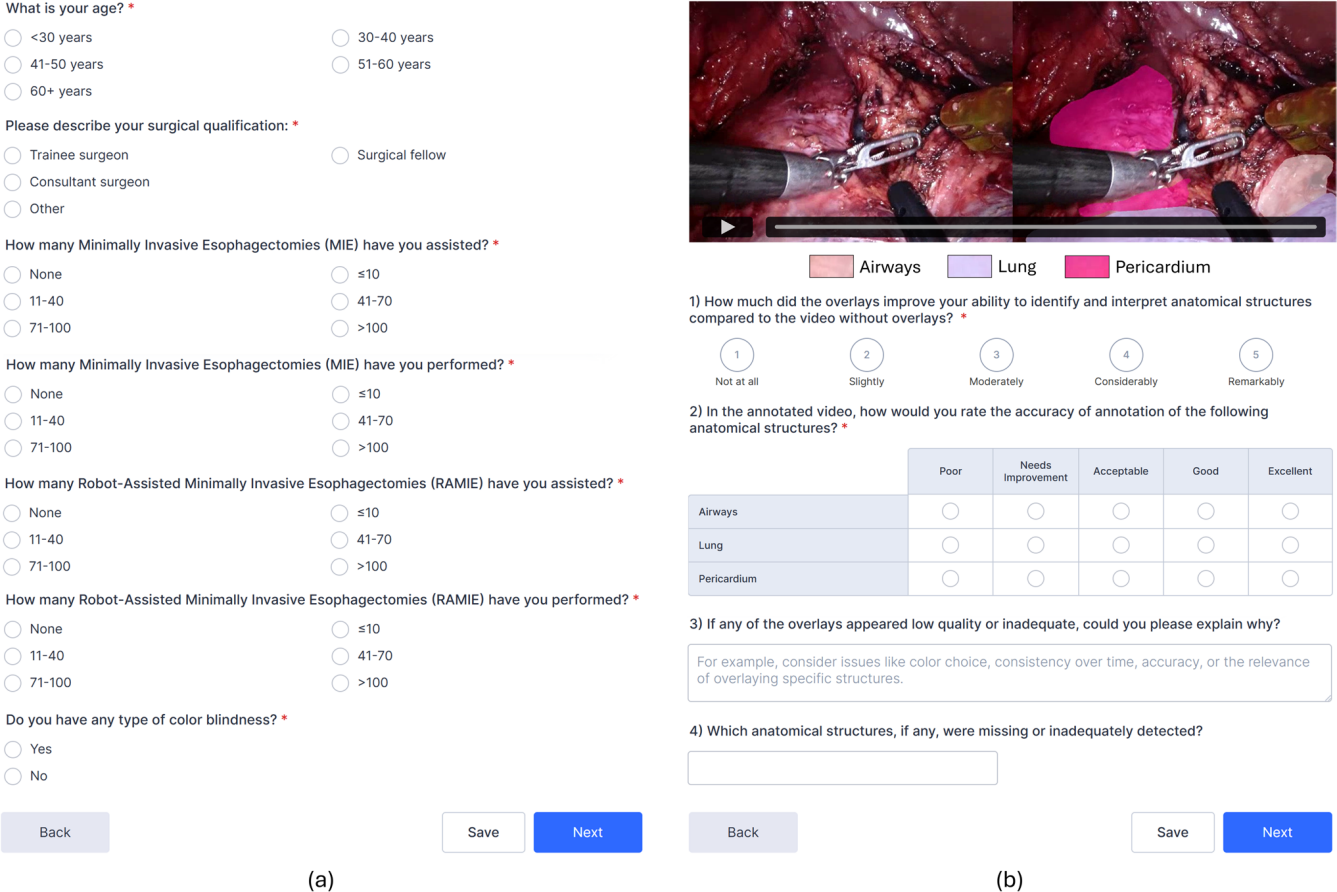
**a** The first section of the survey, in which participants were asked about their demographic and professional background. **b** An example of one of the 12 questions from the second section of the survey. Participants could play the video and answer two compulsory and two optional questions

The survey is divided into two sections, as shown in Fig. 1. The first section is brief and collects key demographic and professional background information from participants, including age, surgical qualifications, number of surgeries performed and assisted, and color blindness. The second section comprises 12 questions, each beginning with a short video clip lasting between 10 and 30 s. Each clip presents a tiled view: The left side shows an unaltered segment of the surgery, while the right side displays the same segment enhanced with overlays generated by a deep learning model. More information on how these videos were selected and how the overlays were generated can be found in the “2.3” section. Before viewing each clip, participants were shown a screenshot from the video without overlays to help them orient themselves and ensure an unbiased evaluation. They then answered four questions per video. The first question assessed whether the overlays improved their ability to identify anatomical structures. The second asked surgeons to rate the accuracy of the overlays for each anatomical structure. Finally, participants were invited to optionally explain why certain overlays were of low quality and to identify any missing or poorly detected structures.

### Survey participants

**Fig. 2:**
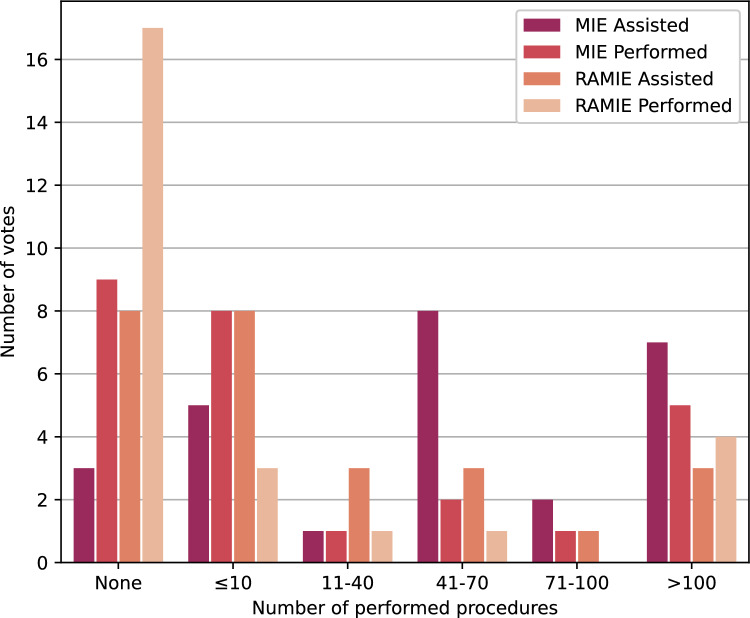
Surgical background of survey participants for minimally invasive esophagectomy (MIE) and RAMIE. Each participant selected one option in each of the four categories shown in the legend, yielding 26 responses per category

The survey was conducted during the ESSO Hands-on Course on Minimally Invasive Gastrectomy and Esophagectomy at the University Medical Center Utrecht, the Netherlands, in November 2024. Prior to the survey, participants received a detailed overview outlining the study’s purpose and procedures. Participation was entirely voluntary, and each person provided informed consent prior to completing the survey. In total, 26 upper gastrointestinal surgeons were included in the survey. None of the participants reported any type of color blindness. On average, participants finished the survey in 10–15 min. An overview of their surgical background is shown in Fig. 2. There is substantial variation among participants in the number of procedures they assisted and performed. We intentionally surveyed individuals with both extensive and limited experience in minimally invasive surgery to assess how their perspectives differ.

### Deep learning models and dataset

In this survey, we utilized overlays generated by two deep learning models. The first model is Mask2Former, pretrained on ADE20K, as used in previous work [13]. Additionally, we employed CAFormer, pretrained on SurgeNet, as proposed in [2]. The training settings were kept consistent with those in the original papers. These models were selected as they represent the best-performing architectures for anatomy recognition in RAMIE reported in the literature. Since the models differ in architecture, using both generates more diverse overlays than a single model would, supporting the goal of exposing surgeons to varied examples.

In order to train the deep learning models, thoracoscopic RAMIE recordings were retrospectively acquired from 32 esophageal cancer patients treated between January 2018 and July 2021 at the University Medical Center Utrecht, The Netherlands. All procedures were performed by two surgeons who had performed over 200 RAMIE cases. Videos were recorded at 25 Hz and 960 × 540 resolution and then cropped to 668 × 502 to remove irrelevant black side borders and portions of the user interface. From these, 879 frames were randomly sampled and annotated by four research fellows under expert surgeon supervision. These frames were split into training (70%), validation (15%), and test (15%) sets based on a per-patient split.

### Overlay generation

Twelve video segments from four patients in the deep learning test set were selected for the survey. These segments were chosen to ensure representation of all target anatomical structures, include both straightforward and more challenging views (e.g., variable lighting, partial occlusion, or anatomical variation), and to sample from multiple patients to avoid patient-specific bias. Each segment ranged from 10 to 30 s, yielding a total of 8326 frames. The following seven anatomical structures are present in these videos: airways, aorta, azygos vein and vena cava, esophagus, nerves, pericardium, right lung, and thoracic duct. The labeling protocol was developed in collaboration with an expert surgeon. The trachea, left, and right main bronchi were grouped into one class due to their similar appearance and indistinct boundaries. The vena cava, azygos vein, and intercostal veins were combined for the same reason. The nerves include the vagal and recurrent laryngeal nerves. Lastly, the pericardium class also encompasses the pulmonary veins, as they lie beneath the pericardial tissue layers. Overall, the survey includes 46 overlays of the aforementioned anatomical structures. These overlays were created using two deep learning models. Half of the predicted video segments were randomly assigned to be generated by Mask2Former, while the other half were generated using CAFormer, ensuring variety in the predicted segments. The predictions were averaged over time to reduce flickering using the following weighted average:

$$\widehat{{F}_{t}}={\sum }_{k=0}^{9}{w}_{k}{F}_{t-k},$$

where *F*_*t*_ denotes the predicted frame at time *t*, $\widehat{{F}_{t}}$ denotes the averaged prediction at time *t*, and the weights are defined as *w*_*k*_ = 1*.*0 − 0*.*1* k*.

**Fig. 3:**
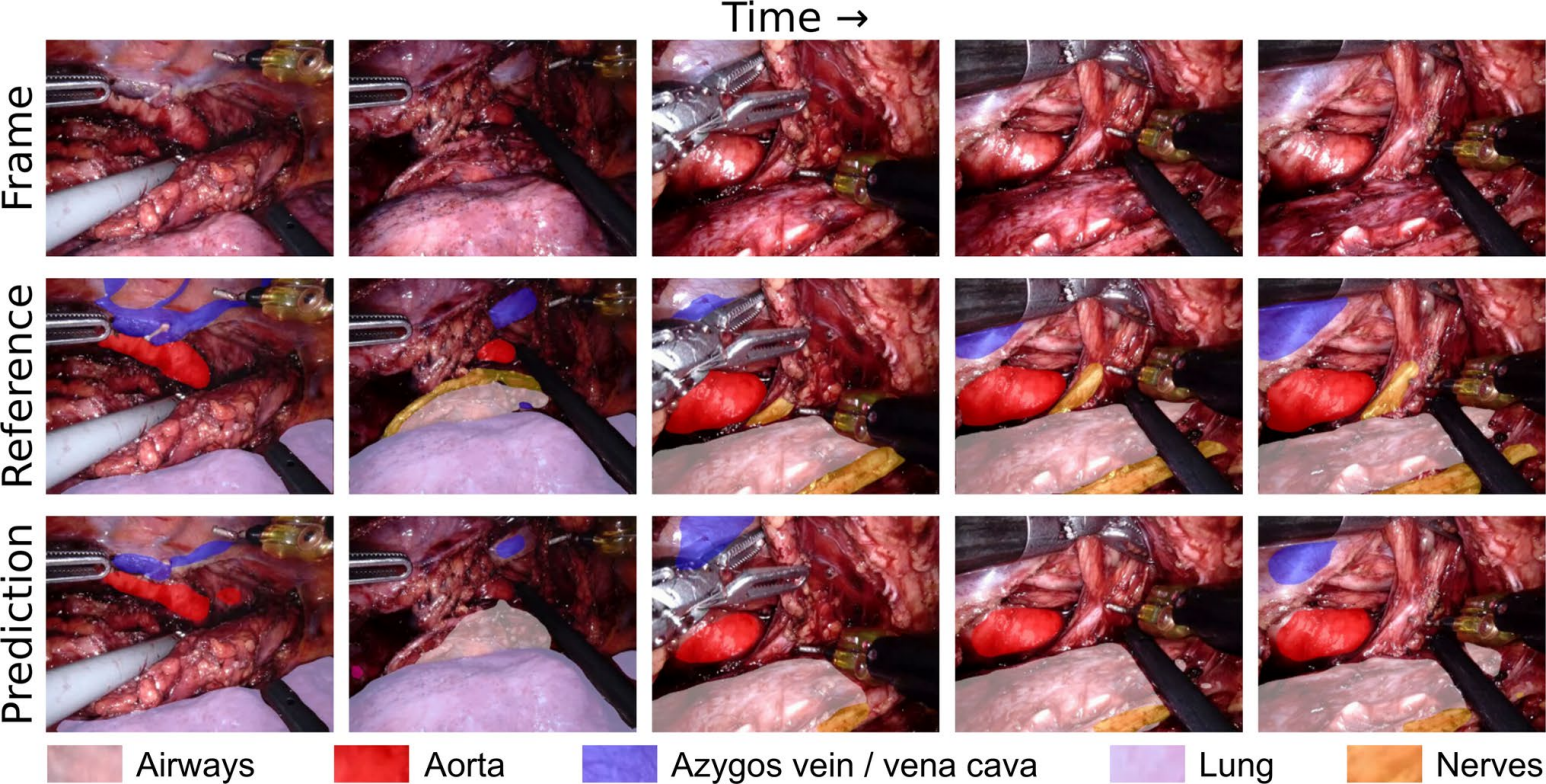
Example of five equidistantly extracted frames from a single video, with reference annotations and model predictions

In order to compute evaluation metrics for each predicted video in the survey, we required reference annotations of all 8326 frames. These reference annotations were created using a combination of RITM [14], a deep learning model for click-based interactive segmentation, and Cutie [15], a deep learning model for temporal propagation of annotations. RITM was fine-tuned on the deep learning dataset for optimal results using the default training settings. Although this method worked reasonably well, all 8326 frames were manually corrected by a research fellow to ensure accurate reference annotations. Figure 3 shows a sample comparison between reference annotations and model predictions from one video.

### Metrics

A comprehensive set of evaluation metrics was included for analysis, with definitions and explanations provided in Appendix A. These metrics are grouped into four categories: (1) Overlap metrics, such as the Dice score, measure how well the predicted segmentation aligns with the reference annotation, which is particularly important for assessing the general accuracy of the overlays. (2) Distance metrics, such as the Hausdorff distance, evaluate the boundary deviation between predicted and actual borders. These could be critical in surgical contexts where even small deviations near vital structures can have clinical implications. (3) Temporal metrics, such as variations in the number of predicted pixels over time, capture the stability and consistency of predictions throughout a video or dynamic sequence. Temporal coherence may be a crucial factor in establishing qualitative trust for surgeons, who benefit from smooth, continuous feedback during procedures. (4) Error-specific metrics, such as the false positive rate, highlight types of mistakes that may carry higher clinical risk, e.g., incorrectly identifying non-critical tissue as critical or vice versa. These metrics support a more nuanced understanding of model behavior and might align better with how surgeons think about safety and reliability.

### Evaluation

The metrics and surgeon ratings of annotation accuracy were compared using the Spearman correlation coefficient, a non-parametric statistic that measures the strength and direction of a monotonic relationship between two ranked variables. Additional explanation and the equation are provided in Appendix B. To facilitate evaluation, we computed the absolute value of the correlation, as some metrics exhibit negative correlations. To investigate the influence of experience in surgery, we have made a specific experience-based split:

1. Experts (> 100 (RA) MIE cases performed) have mastered the complex anatomy, resulting in consistent intra-operative decision-making and accurate anatomical recognition. According to the literature, this number of cases typically surpasses the learning curve for (RA)MIE procedures [4].
2. Intermediates (> 10 (RA)MIE cases performed/assisted; ≤ 100 cases performed) are in the process of refining their anatomical knowledge and technical skills through hands-on and assisted experience.
3. Novices (≤ 10 (RA)MIE cases performed/assisted) are early in their training, having limited familiarity with the anatomy and minimally invasive procedures, which may affect their assessment of anatomical overlays.

Based on this classification, the study participants include 7 experts, 11 intermediates, and 8 novices.

## Results

### Quantitative results

**Fig. 4:**
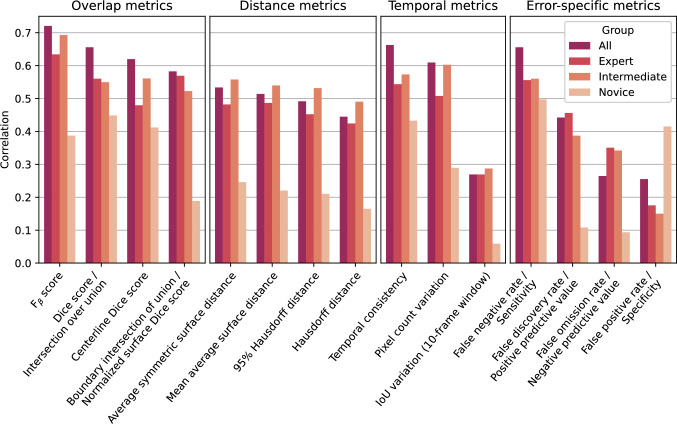
The absolute values of the Spearman correlation coefficients between surgeon accuracy ratings and all metrics, organized by metric type and group. Correlations are computed across 46 samples, where each sample corresponds to an anatomical structure in one of the 12 videos

Figure 4 shows the absolute correlation between various performance metrics and the average surgeon’s ratings of annotation accuracy across all anatomical structures. The results are organized by metric type and stratified by user expertise level. Note that some metrics are combined due to their monotonic relationships, which result in identical Spearman correlation coefficients.

Overlap metrics: Within the overlap metrics, the *F*_*β*_ score exhibits the highest correlations with human ratings, followed by the intersection over union and Dice score. The normalized surface Dice score and the boundary intersection over union are lower overall but remain high in the expert group, suggesting that boundaries are more important for surgeons with more experience.

Distance metrics: Among the distance metrics, correlations are moderate across all groups, with the average symmetric surface distance exhibiting the highest correlation. This suggests that accurate boundary delineation, on average, is more critical than minimizing the maximum boundary error.

Temporal metrics: Some of the temporal metrics show a strong correlation. Specifically, the temporal consistency and pixel count variation correlate better than the IoU variation over a 10-frame window, indicating that high-frequency pixel variations are more disruptive than low-frequency ones.

Error-specific metrics: Error-specific metrics show the greatest variability across the data. Sensitivity and false negative rate have the highest correlation, highlighting the importance of identifying true positives while minimizing false negatives. Interestingly, the perceived scores from novices show a much stronger correlation with false positive rate and specificity than other groups. This suggests that novices are more affected by false positives, possibly due to less prior knowledge compared to experienced surgeons.

In general, novices show lower correlations across most metrics. This is likely due to their limited familiarity with the surgical procedure, which may reduce their ability to critically assess segmentation quality. Additionally, novices tend to give higher ratings on average, which compresses the score range and makes it more difficult to identify strong statistical correlations with the quantitative metrics. Overall, overlap and temporal metrics align most closely with human ratings, particularly among more experienced raters, highlighting their potential as reliable indicators of perceived segmentation quality. Temporal metrics may be particularly relevant because flickering or rapid changes in segmentation can be perceived as disturbing, especially during surgery when distractions are unwanted.

**Fig. 5:**
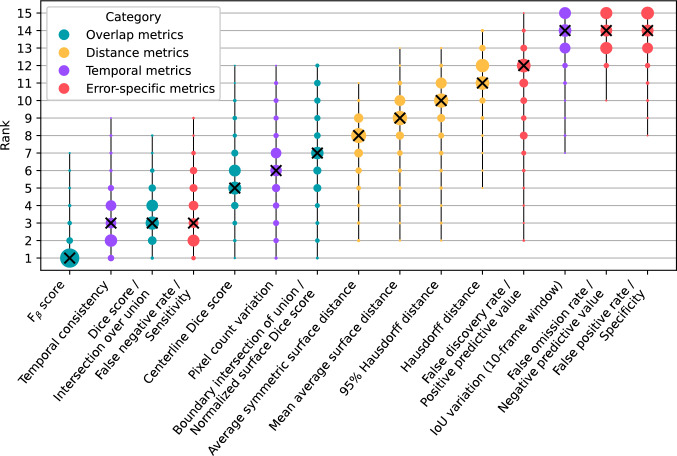
Ranking stability across all metrics obtained by bootstrapping 1000 random samples, each consisting of 35 out of 46 anatomical structures (approximately 75%). Metrics are ordered from left to right, with the best-performing metric on the left and the worst on the right, based on the median rank score across bootstrap samples. In cases where metrics share the same median, the mean rank is used to break ties

While correlation coefficients quantify the overall relation between metrics and surgeon ratings, they do not capture how statistically consistent these relations remain across different subsets of the data. Therefore, we present a ranking stability plot, as shown in Fig. 5, which provides a statistical estimate of how reliably each metric preserves its relative ranking across varying samples. The ranks are mostly in line with the correlations found earlier. However, there is substantial variability in ranks, as indicated by the spread of dots for each metric. This suggests that while the average rankings of the metrics remain generally stable, their relative performance can vary across different data subsets. This variability is likely due to the high diversity of the video clips presented, which affects both metric outcomes and surgeon assessments. Consequently, it is advisable to present as many examples as feasible when conducting survey-based evaluations. However, even presenting only twelve video clips was highly time-intensive for the participants.

### Class-specific results

**Fig. 6:**
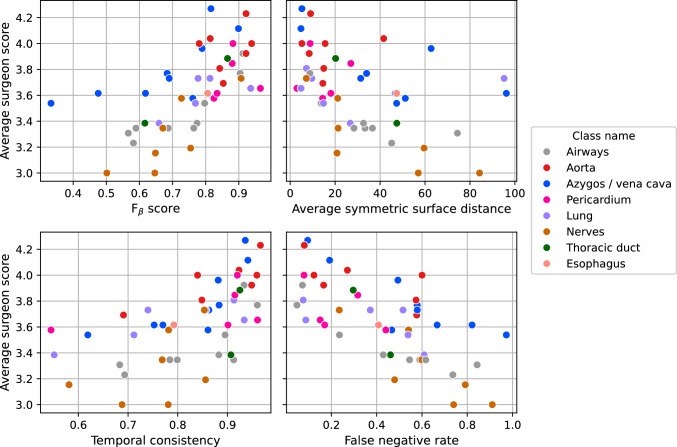
Scatter plot showing average surgeon ratings versus the four best correlating metrics in each category. Each dot represents an overlay of a particular class, indicated by its color

Figure 6 presents scatter plots of surgeon ratings versus the best-correlating metrics in each category. Note that the correlations are negative for average symmetric surface distance and false negative rate, where lower values indicate better segmentation performance. Additionally, distinct patterns emerge across different classes, as indicated by the color-coded dots. Notably, the azygos/vena cava class exhibits substantial deviation. This may be attributed to the azygos vein appearing as a small peripheral feature in some videos, potentially leading to lower metric scores despite high ratings from surgeons. It can also be observed that some classes appear much more frequently than others. The esophagus is included only once in the survey, consistent with prior surgeon feedback indicating it is not a primary structure of interest, largely because it is less relevant to intra- and postoperative complications.

**Fig. 7:**
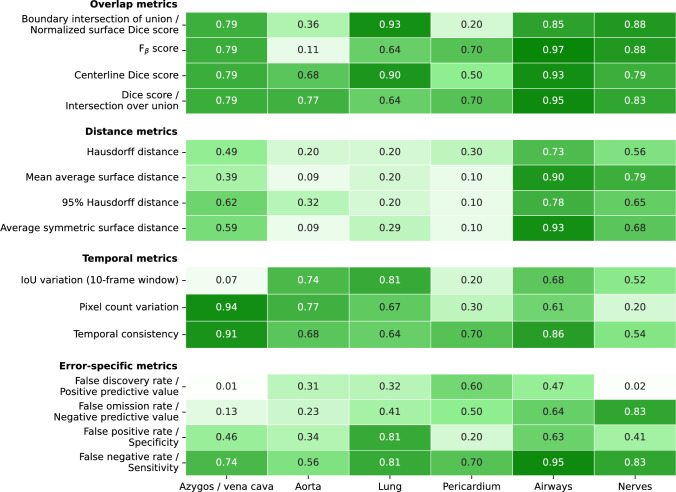
Absolute value of Spearman correlation coefficient for all metrics organized per metric type and anatomical structure

Figure 7 presents the correlation per anatomical structure, offering a more detailed view of class-specific patterns. While this analysis provides finer-grained insights, it is also inherently less robust due to the reduced sample size. This is because metrics are averaged over instances within a single class. This reduced sample size also explains why all overlap metrics have the same score in the azygos/vena cava class. The esophagus and thoracic duct are excluded from the analysis, as they have only one and two samples, respectively, resulting in artificially perfect correlations. Despite these limitations, Fig. 7 shows that the strongest correlations occur in the airways and nerves. This may be attributed to the larger number of samples with varying segmentation quality in these classes, which facilitates the detection of statistical relationships. Another plausible explanation is that these structures receive more focused attention during surgery, given their clinical significance and the potential complications associated with injuring them. In contrast, correlations in the other classes are less apparent, likely due to uniformly higher segmentation quality, which reduces observable variability and thus limits the potential for correlation.

### Usefulness and qualitative feedback

While we observe substantial correlations between objective metrics and the surgeons’ assessments of annotation accuracy, this does not necessarily imply improved ability to identify and interpret anatomical structures. However, we additionally find a strong Pearson correlation coefficient (0.77) between the average responses to question one (regarding identification ability) and question two (regarding annotation accuracy), suggesting that higher annotation accuracy likely supports better anatomical interpretation. Nevertheless, it is important to note that this finding is based on a limited sample of 12 videos.

Several respondents noted that lymph nodes and aorto-esophageal branches are missing from the videos, emphasizing the importance of aligning the classes used in the guiding algorithm with current clinical needs. The respondents also identified multiple limitations regarding the quality and anatomical accuracy of the overlays. Common concerns include fluctuating or unstable segmentations, which align with the high correlation observed in the temporal consistency metric. Overlays extending over dissection planes are also frequently mentioned, highlighting the importance of incorporating distance metrics.

**Fig. 8:**
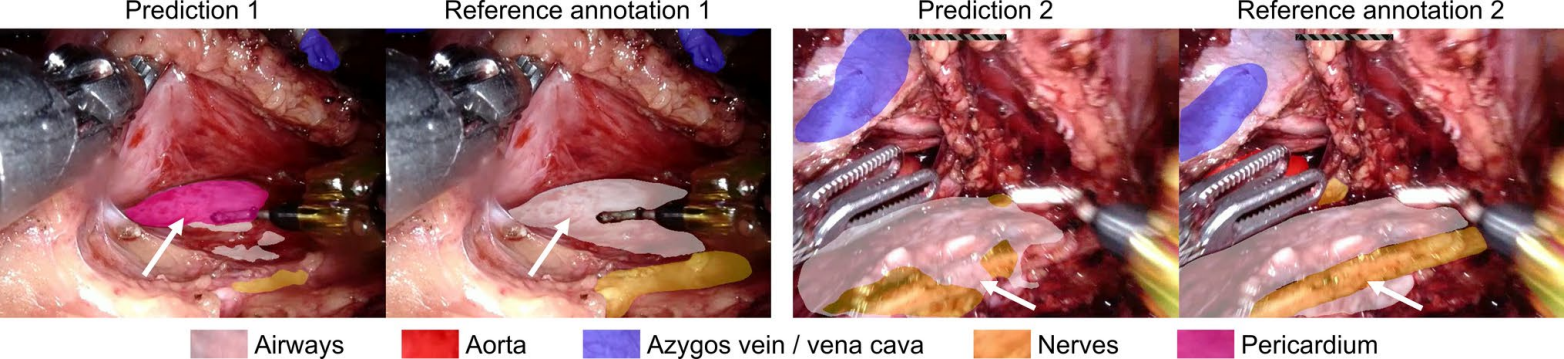
Left: Prediction and reference annotation showing the airways misclassified as pericardium. Right: Prediction and reference annotation showing partial misclassifications between nerves and airways

Anatomical inaccuracies often involve structures such as the airways, nerves, thoracic duct, and pericardium. These are frequently described as missing, mislabeled, or incompletely highlighted. Some respondents also reported hallucinations in structure identification, such as the airway being labeled as the pericardium or a nerve. Examples of such cases are shown in Fig. 8. Interestingly, our metric evaluation does not reflect these false positives with high correlation. One possible explanation is that even very short misclassifications can significantly affect surgeon ratings, while their impact on average metrics over time remains minimal.

## Discussion

This study provides a comprehensive evaluation of the clinical relevance of segmentation metrics through direct input from surgeons. By correlating standard quantitative performance metrics with expert evaluations in several RAMIE video clips, we aim to bridge a critical gap between algorithmic development and practical utility in the operating room.

Our findings demonstrate that traditional segmentation metrics—particularly overlap-based metrics such as the Dice score and intersection over union—exhibit strong correlations with surgeon ratings, especially among experienced participants. This suggests that these metrics, despite their algorithmic simplicity, do capture meaningful aspects of perceived anatomical accuracy in the context of complex minimally invasive surgeries. However, other commonly used metrics, such as Hausdorff distance, show less consistent alignment with surgeon assessments, highlighting potential limitations in their ability to reflect clinically relevant segmentation quality. Nevertheless, it is important to select metrics from different categories to ensure a comprehensive evaluation, avoiding redundancy where multiple metrics capture the same aspects of performance. Furthermore, textual feedback reveals the importance of accurate boundary delineation.

Importantly, our experience-based stratification reveals clear patterns in metric relevance. Experts demonstrate stronger and more consistent correlations across multiple metric types compared to novice participants. This likely reflects their ability to recognize anatomical structures faster and more accurately. Conversely, novice raters appear less sensitive to segmentation quality and more influenced by the presence of any anatomical guidance, which may explain their generally higher and less variable ratings. These findings highlight the importance of considering user expertise when validating deep learning-based anatomy segmentation models.

Temporal consistency metrics also exhibit substantial correlations with surgeon feedback, which aligns with qualitative comments indicating visual instability and flickering as distracting. This highlights the potential utility of incorporating temporal smoothness as a standard evaluation criterion in video-based anatomical segmentation tasks. Similarly, qualitative feedback emphasizes the negative impact of structural mislabeling—even when occurring for short periods of time—on clinical trust and usability. Notably, such issues are not always captured effectively by the selected error-specific metrics. This discrepancy suggests that future metric development may benefit from incorporating temporally weighted error penalties or spatial prioritization strategies that better reflect real-time surgical impact. Furthermore, the metrics evaluated in this paper were developed to quantify segmentation accuracy, not to assess broader aspects of surgical quality. As such, important elements of technique, such as traction, counter-traction, or plane development, are not represented. This gap highlights the need for future surgeon-informed metrics that better capture clinically relevant aspects of performance.

Class-specific analysis reveals that metric-surgeon alignment varies across anatomical structures. Strong correlations are observed for nerves and airways, which are both clinically critical and prone to variable appearance during RAMIE. These findings suggest that correlation coefficients may be more reliable for structures where segmentation quality is more heterogeneous and where small errors carry greater clinical weight. Conversely, structures with uniformly high segmentation quality, such as the aorta, show less correlation, not necessarily due to metric inadequacy but likely due to a compressed range of performance scores. These insights emphasize the need for stratified or structure-aware evaluation strategies in future work.

Several limitations of our study should be acknowledged. First, the sample size and the number of evaluated structures per class are limited by practical constraints related to participant time and attention. Second, the resolution of our videos is lower than the 4 K quality now available in some systems. Lower resolution may have reduced boundary sharpness, particularly for small or thin structures, potentially affecting both segmentation performance and surgeon ratings. However, this resolution still reflects common clinical recording practice in RAMIE. Third, our focus on retrospective video analysis, while enabling controlled evaluation, does not fully replicate the cognitive demands of live surgery. Real-time usability studies may yield additional insights into the clinical utility of deep learning overlays. Moreover, some surgeons commented on aspects such as image size, color choices, and overlay opacity, which may have influenced their responses. Future studies should better clarify that the goal is to assess overlay accuracy rather than the style of presentation. Additionally, not all participants understood our annotation of the thoracic duct, as we included the surrounding fatty tissue. We therefore received multiple comments regarding the mislabeling of this structure. Future surveys should explicitly explain such annotation subtleties.

Furthermore, generating reference annotations is inherently difficult, and it is unclear whether they fully align with surgeons’ expectations. Future work should consider presenting reference annotations as well to better understand their validity. Finally, our evaluation is limited to RAMIE procedures. To more comprehensively assess the utility of deep learning-based overlays in surgery, future research should include a broader range of surgical procedures.

In conclusion, this study highlights the value of combining quantitative metrics with qualitative surgeon input to evaluate deep learning–based anatomical segmentation. Overlap metrics show strong alignment with clinical assessments, while temporal and distance-based metrics are useful for detecting visual stability and boundary precision. However, certain error types such as short misclassifications remain underrepresented in current evaluation frameworks. Our findings support the continued use of standard metrics but emphasize the need to augment them with surgeon-informed evaluations and task-specific considerations. A practical framework could combine (i) standard quantitative metrics for reproducibility, (ii) surgeon-in-the-loop evaluations through structured video reviews to capture issues like hallucinations or instability, and (iii) task-specific metrics that weight errors near critical structures or penalize temporal inconsistencies. This layered approach could be introduced in clinical practice in an “observer mode,” where overlays are displayed but not used for decision-making. This allows for systematic feedback and safe, iterative refinement toward real-world integration. Together, these strategies can ensure that AI-guided surgical tools not only perform well numerically but also provide meaningful, clinically relevant support during surgery.

## Appendix A: Evaluation metrics

This section describes the metrics used to evaluate segmentation performance. The metrics are grouped into four categories: overlap, distance, temporal, and error-specific metrics. Apart from the temporal metrics, all metrics are calculated frame-by-frame and then averaged per class and video clip. Throughout this section:

- *A*: predicted segmentation
- *B*: reference annotation
- *A*_*b*_, *B*_*b*_: boundaries of *A* and *B*
- *A*_*cl*_, *B*_*cl*_: centerlines of *A* and *B*
- *d*(*x,Y*): shortest Euclidean distance from point *x* to set *Y*
- *TP*: true positives
- *FP*: false positives
- *FN*: false negatives
- *TN*: true negatives

### A.1 Overlap metrics

Overlap metrics evaluate how much the predicted segmentation *A* overlaps with reference annotation *B*. Higher values indicate better overlap.

#### A.1.1 Dice score

The Dice score measures the spatial overlap between prediction and reference:

$${\mathrm{Dice}}=\frac{2\left|A\cap B\right|}{\left|A\right|+\left|B\right|}$$

#### A.1.2 Intersection over union (IoU)

Also known as the Jaccard Index, IoU quantifies how well the predicted region matches the reference:

$${\mathrm{IoU}}=\frac{\left|{\boldsymbol{A}}\cap {\boldsymbol{B}}\right|}{\left|{\boldsymbol{A}}\cup {\boldsymbol{B}}\right|}$$

#### *A.1.3 F*_*β*_* score*

The *F*_*β*_ score balances precision and recall, with *β* controlling the importance of recall:

$${F}_{\beta }=\frac{\left(1+{\beta }^{2}\right)\cdot \mathrm{TP}}{\left(1+{\beta }^{2}\right)\cdot \mathrm{TP}+{\beta }^{2}\cdot \mathrm{FN}+\mathrm{FP}}$$

In this research, we set *β* to 0*.*5.

#### A.1.4 Boundary intersection over union

This metric evaluates the overlap between the boundaries of the predicted and reference masks:

$$\text{Boundary IoU}=\frac{\left|{A}_{b}\cap {B}_{b}\right|}{\left|{A}_{b}\cup {B}_{b}\right|}$$

#### A.1.5 Centerline dice score

This measures agreement of the centerlines of predicted and reference structures:

$$\text{Centerline Dice}=\frac{2\left|{A}_{\mathrm{cl}}\cap {B}_{\mathrm{cl}}\right|}{\left|{A}_{\mathrm{cl}}\right|+\left|{B}_{\mathrm{cl}}\right|}$$

#### A.1.6 Normalized surface dice score

Normalized surface dice score assesses surface agreement between *A* and *B*, normalized by surface area:

$${\mathrm{NSD}}=\frac{2\left|{{\boldsymbol{A}}}_{{\boldsymbol{b}}}\cap {{\boldsymbol{B}}}_{{\boldsymbol{b}}}\right|}{\left|{{\boldsymbol{A}}}_{{\boldsymbol{b}}}\right|+\left|{{\boldsymbol{B}}}_{{\boldsymbol{b}}}\right|}$$

### A.2 Distance metrics

Distance metrics quantify how far apart the surfaces of the predicted and reference segmentations are. Lower values indicate better surface alignment.

#### A.2.1 Average symmetric surface distance (ASSD)

Average symmetric surface distance (ASSD) computes the average distance from the surface of *A* to *B*, and vice versa:

$${\mathrm{ASSD}}=\frac{{\sum }_{{\boldsymbol{a}}\in {{\boldsymbol{A}}}_{{\boldsymbol{b}}}}{\boldsymbol{d}}\left({\boldsymbol{a}},{{\boldsymbol{B}}}_{{\boldsymbol{b}}}\right)+{\sum }_{{\boldsymbol{b}}\in {{\boldsymbol{B}}}_{{\boldsymbol{b}}}}{\boldsymbol{d}}\left({{\boldsymbol{A}}}_{{\boldsymbol{b}}},{\boldsymbol{b}}\right)}{\left|{{\boldsymbol{A}}}_{{\boldsymbol{b}}}\right|+\left|{{\boldsymbol{B}}}_{{\boldsymbol{b}}}\right|}$$

#### A.2.2 Mean average surface distance (MASD)

Mean average surface distance (MASD*) *calculates the mean surface distance by averaging separately for each surface:

$${\mathrm{MASD}}=\frac{1}{2}\left(\frac{{\sum }_{a\in {A}_{b}}d\left(a,{B}_{b}\right)}{\left|{A}_{b}\right|}+\frac{{\sum }_{b\in {B}_{b}}d\left({A}_{b},b\right)}{\left|{B}_{b}\right|}\right)$$

#### A.2.3 Hausdorff distance

Hausdorff distance reports the largest surface-to-surface distance, capturing worst case deviation:

$${\mathrm{HD}}=\mathrm{max}\left\{\underset{a\in {A}_{b}}{\mathrm{max}}d\left(a,{B}_{b}\right),\underset{b\in {B}_{b}}{\mathrm{max}}d\left({A}_{b},b\right)\right\}$$

#### A.2.4 95% Hausdorff distance

A robust version uses the 95th percentile of distances to reduce sensitivity to outliers:

$${\mathrm{HD}}_{95}=\mathrm{max}\{{d}_{95}\left({A}_{b},{B}_{b}\right),{d}_{95}\left({B}_{b},{A}_{b}\right)\}$$

### A.3 Temporal metrics

Temporal metrics assess the consistency of segmentation across sequential frames in time-series data. Let *A*_*t*_ denote the prediction at time *t*, and *W*(*A*_*t*−1_) denotes the warped version of *A*_*t*−1_ aligned to time *t*.

#### A.3.1 Temporal consistency

Temporal consistency measures temporal overlap between the warped previous prediction and current prediction. Warping is calculated based on the original frames using optical flow as described by Farneback et al. [16]. This transformation is then applied to the segmentation to obtain a warped version.

$${\mathrm{TC}}=\frac{1}{T-1}{\sum }_{t=2}^{T}\frac{\left|\mathcal{W}\left({A}_{t-1}\right)\cap {A}_{t}\right|}{\left|\mathcal{W}\left({A}_{t-1}\right)\cup {A}_{t}\right|}$$

#### A.3.2 Pixel count variation

Pixel count variation quantifies pixel-level differences between consecutive frames. A key limitation of this metric is that it does not account for moving structures, as in the temporal consistency metric. Nevertheless, it can provide insight into temporal variations:

$${\mathrm{PCV}}=\frac{1}{T-1}{\sum }_{t=2}^{T}\frac{{\sum }_{i}{\sum }_{j}\left|{A}_{t}\left(i,j\right)-{A}_{t-1}\left(i,j\right)\right|}{{\sum }_{i}{\sum }_{j}{A}_{t}\left(i,j\right)}$$

#### A.3.3 IoU variation (10-frame window)

IoU variation (10-frame window) assesses the temporal stability of the IoU across a sliding window of size *w*:

$${\mathrm{IoUVar}}=\frac{1}{T-w+1}{\sum }_{t=w}^{T}\sqrt{\frac{1}{w}{\sum }_{i=t-w+1}^{t}{\left({\mathrm{IoU}}_{i}-\frac{1}{w}{\sum }_{j=t-w+1}^{t}{\mathrm{IoU}}_{j}\right)}^{2}}$$

### A.4 Error-specific metrics

These metrics are based on the confusion matrix and analyze specific classification errors. Let:

- TP (true positives): pixels correctly predicted as foreground
- FP (false positives): pixels incorrectly predicted as foreground
- FN (false negatives): pixels incorrectly predicted as background
- TN (true negatives): pixels correctly predicted as background

#### A.4.1 Sensitivity (recall)

Sensitivity (recall) measures the proportion of actual positives correctly identified:

$${\mathrm{Sensitivity}}=\frac{\mathrm{TP}}{\mathrm{TP}+\mathrm{FN}}$$

#### A.4.2 Specificity

Specificity measures the proportion of actual negatives correctly identified:

$${\mathrm{Specificity}}=\frac{\mathrm{TN}}{\mathrm{TN}+\mathrm{FP}}$$

#### A.4.3 False positive rate (FPR)

The proportion of negatives incorrectly predicted as positives:

$${\mathrm{FPR}}=\frac{\mathrm{FP}}{\mathrm{FP}+\mathrm{TN}}$$

#### A.4.4 False negative rate (FNR)

The proportion of positives incorrectly predicted as negatives:

$${\mathrm{FNR}}=\frac{\mathrm{FN}}{\mathrm{FN}+\mathrm{TP}}$$

#### A.4.5 Positive predictive value (precision)

The proportion of predicted positives that are true:

$${\mathrm{PPV}}=\frac{\mathrm{TP}}{\mathrm{TP}+\mathrm{FP}}$$

#### A.4.6 Negative predictive value

The proportion of predicted negatives that are true:

$${\mathrm{NPV}}=\frac{\mathrm{TN}}{\mathrm{TN}+\mathrm{FN}}$$

#### A.4.7 False discovery rate

The proportion of false predictions among all positive predictions:

$${\mathrm{FDR}}=\frac{\mathrm{FP}}{\mathrm{FP}+\mathrm{TP}}$$

#### A.4.8 False omission rate

The proportion of false predictions among all negative predictions:

$${\mathrm{FOR}}=\frac{\mathrm{FN}}{\mathrm{FN}+\mathrm{TN}}$$

## Appendix B: Spearman correlation coefficient

The results in this paper are evaluated using the Spearman correlation coefficient, defined as:

$$\rho =1-\frac{6{\sum }_{i=1}^{n}{d}_{i}^{2}}{n\left({n}^{2}-1\right)},$$

where *d*_*i*_ is the difference between the two ranks of the *i*th data point, and *n* is the total number of data points. The coefficient measures how well the relationship between two variables can be described by a monotonic function. A value of 1 indicates a perfect positive monotonic correlation, while − 1 represents a perfect negative monotonic correlation.
